# 

**Published:** 1871-01

**Authors:** 


ORIGINAL CONTRIBUTIONS.
The Administration of Emetics during Pregnancy, and their therapeutic Effects in Cases of Threatened Abortion, Miscarriagə, and Premature Labor. By J. G. Stokes, M.D., Grayville, Ill. 1
Tobacco. Read before the Chicago Medical Society by F. A. Emmons, M.D., 8
Cases from the Note Book Robert Robson, M.D., New Harmony Ind.,........ 13

THE CLINIC.
Chronic Hydrocephalus. Clinc by Prof. N. S. Davis, ........................... 21
Sub-Acute Rheumatism. Clinic By Prof. N. S. Davis,............................ 25

CORRESPONDENCE.
Diphtheria. By D. J. McMillan,........ 27
Science vs. the Senses. By Justice,..... 30

SELECTIONS.
Death from Chloroform,.................. 26
Syphilis of the Nervous System,......... 33
Heart Disease and Sea Winds, ........... 34
Simple Dressing by Continued Moisture, 35
Intussusception in an Infant cured by Inflammation of the Bowel,.............. 36
The cause of Dr. Simpson's Death,....... 37
Detection of the Adulteration of Quinine with Salicine, ........................... 38
Tincture of Iron in Acute Rheumatism, . 38
Stramonium Poisoning—Antidotal Power of Opium,.............................. 39
Air in Wounds Innocuous,............... 39
Death by Chloroform Prevented by Electricity,.................................. 40

ABRIDGMENTS FROM EXCHANGES.
Observations on Relapsing Fever as it Occurred in Philadelphia in 1869-70,..... 40
Skin Grafting,.............................. 42
Syphilis of the Nervons System,.......... 44
Hair as Suture and Ligature,.............. 45
Minnesota as a Resort for Consumptives 46
Liquid Glass for Stiff Bandages in Fractures,..................................... 46
Effects of Alcohol on the Human System 47
Large Doses of Ipecacuanha in acute Dysentery,.................................... 48
Partial Paralysis from Reflex Irritation,. 48

BOOK NOTICES.
The American Practitioner: A Monthly Journal of Medicine and Surgery. By David W- Yandell, M.D., and Theophalus Parvin, M.D., ........................ 49
A Tabular History and Analysis. By J. Baxton Upham, A.M., M.D.,........... 49
Surgical Papers of the Transactions of the American Medical Association, 1870 50
Annual Report of the Surgeon-General United States Army,.................... 55
Transactions of the Twentieth Anniversary Meeting of the Illinois State Medical Society, ..................................... 57
Urea is Found in the Liver,.................. 58

EDITORIAL.
American Medical Association,............... 58
Ophthalmology and Otology,.................. 60
Correction, ....................................... 60
Mortality for the Month of November,1870, 61
When to Give Artificial Food to Infant. 62
Catheterism of the Larynx,.................. 62
Money Receipts,............................... 63

